# Trial of improved practices approach to explore the acceptability and feasibility of different modes of chlorhexidine application for neonatal cord care in Pemba, Tanzania

**DOI:** 10.1186/s12884-015-0760-4

**Published:** 2015-12-28

**Authors:** Usha Dhingra, Sunil Sazawal, Pratibha Dhingra, Arup Dutta, Said Mohammed Ali, Shaali Makame Ame, Saikat Deb, Atifa Mohammed Suleiman, Robert E. Black

**Affiliations:** Department of International Health, E5521, Johns Hopkins Bloomberg School of Public Health, 615, North Wolfe Street, Baltimore, MD 21205-203 USA; Public Health Laboratory-Ivo de Carneri, Wawi, Chake-Chake, Pemba, Zanzibar Tanzania; Center for Public Health Kinetics, New Delhi, India

**Keywords:** Trials of Improved Practices (TIPs), Chlorhexidine, Cord care

## Abstract

**Background:**

Infections are responsible for 30–40 % of 4 million neonatal deaths annually. Use of chlorhexidine (CHX), a broad-spectrum topical antiseptic with strong residual activity, for umbilical cord cleansing has been shown to reduce infections during the neonatal period. However, the challenge remains with regard to selection of best mode of CHX delivery. As a part of formative research, we undertook a qualitative study in Pemba Island as a pilot to explore the attitudes; beliefs and practices of the community and health workers related to delivery, newborn and cord care. During the second phase of formative research, we used Trials of Improved Practices (TIPs) methodology to explore the acceptance and impediments, for the three possible modes of chlorhexidine application- 100 ml bottle with cotton swab, 10 ml single use dropper bottle and 3 g single application squeeze tube containing gel, as an umbilical cord care intervention.

**Methods:**

In this pilot study, 204 mother-newborn pairs were enrolled from hospital and community setting in Pemba, Tanzania using a randomized three period crossover design. Mothers/guardians, Trained Birth Attendants (TBA)/ medical staff and community health workers (CHWs) were requested to try three different modes of CHX application for cord cleaning. All participants were demonstrated the method of cord cleaning using all three modes of delivery; each delivery mode was used for 3 days and an interview was conducted on day 10 to collect summary of their experience. Acceptance and preference scores were calculated based on feedback from the participants.

**Results:**

Of 204 mother-newborn pairs, 27 were lost to follow up. 177 mothers performed the intervention and applied CHX to the newborn cord for all 9 days. Mothers rated 10 ml dropper bottle (49.7 %) as most convenient in terms of ease and application. They selected 10 ml dropper bottle (44.6 %) as their first choice; gel tube (33.9 %) and 100 ml bottle (21.5 %) as their second and third choice. TBAs, medical staff and CHWs also preferred 10 ml dropper bottle (43.3 %) over 100 ml bottle (12.9 %) and gel (38.8 %).

**Conclusions:**

Overall acceptability of CHX application for cord cleansing was high. 10 ml single use dropper bottle was given highest preference for CHX application. An understanding of the attitudes, beliefs and cultural practices in the community and selection of the most acceptable mode of CHX delivery is essential to the design and implementation of the intervention trials examining the efficacy of CHX cord care in reducing neonatal mortality and subsequent implementation in the programs.

**Trial registration:**

ClinicalTrials.gov NCT01528852 Registered February 3, 2012

## Background

Neonatal mortality accounts for 70 % of deaths in the first year and 40 % of total under-five mortality [1–3]. Each year nearly 4 million children die before 4 weeks of age globally, of which over 1.1 million neonatal deaths (28 %) occur in sub-Saharan Africa. Infections are responsible for 30–40 % of 4 million neonatal deaths annually [1, 4–6]. Omphalitis, an infection of the umbilical stump, resulting from colonization of the stump with bacteria from the maternal genital tract and the environment poses a significant risk of infection and death during the first 28 days of life. Effective interventions that can be carried out at the household level are critically needed to reduce neonatal infections and mortality.

WHO recommends clean and dry cord care for newborns born in health facilities, and at home in areas with low neonatal mortality rates (<30 per thousand). However, they also propagate application of topical antiseptics to the cord stump during the first week of life - for home deliveries in areas where the risk of bacterial infection appears high (30 or more neonatal deaths per 1000 live births) [7]. Chlorhexidine is a broad-spectrum topical antiseptic with strong residual activity. CHX has shown a potential to reduce infections during the neonatal period [8, 9]. Being inexpensive, along with a strong safety profile, CHX seems to be an ideal antiseptic for cord care in low-resource communities [10, 11]. In 2013, WHO added 7.1 % chlorhexidine digluconate (delivering 4 % chlorhexidine) to its list of essential medicines for children [12]. Community level randomized controlled trials in Nepal, Pakistan, and Bangladesh have shown that applying a 4 % chlorhexidine product (7.1 % chlorhexidine digluconate) to the umbilical cord has the potential to save lives [13–15]. Two studies in Nepal tested both aqueous and gel chlorhexidine formulation and observed that gel formulation was more acceptable and preferred than liquid solution [16, 17]. Use of 4 % chlorhexidine umbilical cord wash as a low-cost intervention can easily be scaled up and incorporated into preventive health care in sub-Saharan Africa, impacting a part of the 1.1 million neonatal deaths and 27 million years of life lost every year in sub-Saharan Africa [18–21].

Given the promising results from recent chlorhexidine research and an understanding of the existing practices and beliefs related to newborn care, feasibility of implementing a liquid cleansing solution and selection of the most acceptable mode of delivery of intervention are essential to the design and implementation of intervention trials examining the efficacy of use of chlorhexidine to clean umbilical cord of neonates in sub-Saharan Africa and also for implementation of programs if found efficacious.

We carried out a formative research phase before starting of the main efficacy trial. In phase 1 of formative research, Focus group discussions (FGD’s) and in-depth interviews were held to understand the neonatal care and umbilical cord care perceptions and practices in the community; and evaluate the acceptance and barriers regarding the use of proposed chlorhexidine cleansing solution. In the second phase, Trial of Improved Practices (TIPs) methodology was used to ascertain the acceptability and preference for various possible modes of chlorhexidine delivery for cord care among the mothers/caretakers and health professionals. TIPs is developed by Manoff group, and is a formative research method that engages potential participants in the design of program strategies and activities focused on behavior change, wherein participants try new practices as part of their routine over a trial period; and then provide feedback at the end of the trial period [22]. The results of phase-1 have already been published elsewhere [23] and in this paper we are reporting the findings of the Phase-2 of the formative research. The findings from this phase would be useful in the design and implementation of a culturally acceptable intervention for a large double-blind community-based randomized controlled trial evaluating the impact of chlorhexidine cord cleansing in first 10 days for reduction in omphalitis and neonatal mortality in Pemba, Tanzania where the signs of omphalitis appear frequently and predominantly in the first week of life among newborns [24]. In Asia, Alam et al. [25] adopted a similar strategy and carried out a formative research study in Sylhet, Bangladesh to assess the umbilical and skin care knowledge and practices for neonates in preparation for a cluster-randomized trial of the impact of topical chlorhexidine cord cleansing on neonatal mortality and omphalitis. Our pilot study will contribute to the design of programs intending to implement chlorhexidine cord care interventions in Africa and elsewhere.

## Methods

### Study area and subjects

The study was carried out in Pemba Island, Tanzania, the smaller of the two islands of the Zanzibar archipelago. All births occurring in October and November 2010 at five major hospitals (four district hospitals and one cottage hospital) in the island and in the community were included in the study till the desired sample size was achieved.

### Ethical approval

Ethical approval for the study was obtained locally from the Zanzibar Medical Research and Ethics Committee (ZAMREC) and from the Johns Hopkins Bloomberg School of Public Health Committee on Human Research. Informed verbal consent was obtained from all study participants in local language.

### Study procedures

From the community and maternity wards of the hospitals, 204 mother-newborn pairs were enrolled to pretest three possible modes of intervention i.e. 100 ml bottle with cotton swab (A), 10 ml single use dropper bottle (B) and 3 gm single application gel tube (C) using Trials of Improved Practices (TIPs) methodology. A communication network was established with all the trained birth attendants (TBAs), maternal and child health (MCH) workers, hospital staff and health professionals working on the island. Each personnel was provided with a cell phone and a 24-hour study call center was set up at the central office to ensure immediate and regular communication.

### Data collection tools

The Research Scientists in consultation with the Principal Investigator designed the working protocol, methods for data collection, standard operating procedures (SOPs) and consent forms for study implementation. Detailed questionnaires were prepared for collection of socio-demographic information, pregnancy history, birth characteristics, and newborn care practices at enrollment, compliance information at follow up visits and participant feedback at the evaluation visit. Log sheets were designed to help mothers record CHX application on daily basis by simply putting a tick mark.

### Training and reliability

Training sessions were organized to train the MCH staff, TBAs, hospital staff and health workers on application of all the three modes of intervention. This was followed by practice session wherein each one of them practiced CHX application on a dummy. A dry run was conducted to ensure reliability and effective implementation of study protocol. On scheduled visits, mothers were demonstrated and instructed to apply chlorhexidine to the tip (over the cut surface) of the cord, the stump and around the base of the stump.

### TIPs intervention

First phase of formative research (ethnography) helped us to understand the barriers and facilitators to the introduction of chlorhexidine as a cord care regimen, develop communication messages, study procedures and the framework for implementing a cord care intervention based on the information gathered. TIPs phase involved initial enrollment visit, two follow up visits and a final assessment visit. Study team demonstrated the use of different containers for CHX application to the mother at the enrollment and follow up visits. Essential newborn and cord care messages were given to the mothers at each visitation. Mother’s feedback about different containers was recorded in a standard questionnaire.

### Randomization

Two separate randomization schedules were generated for hospitals and community births. There were 6 possible sequences of allocating the enrolled mother-newborn pair to one of the 3-intervention modes- 100 ml bottle (A), 10 ml dropper bottle (B) or gel tube (C). A mother-newborn pair randomized to sequence 1 (A B C) would use 100 ml bottle for first 3 days, 10 ml single use dropper bottle for next 3 days and get tube for the last 3 days. Other Possible sequences were (A C B), (B A C), (B C A), (C A B) or (C B A). Each delivery mode was used for the same period (three consecutive days). Randomization was done using permuted block randomization method (block length of 12) which generated a list of randomly allocated intervention sequence against a serial number. This ensured uniform distribution of the intervention sequence (three application methods) to one of the six possible delivery sequences. Envelopes were prepared with serial number written on them and the assigned intervention sequence sealed inside the envelope. Upon enrollment, the supervisor opened the next envelope from the sequence and allocated the enrolled mother-newborn pair to the intervention sequence/pack printed inside it and applied the first application mentioned in the slip to the child. Until the opening of the seal of the envelope, both the supervisor/researcher and the mothers were kept blind to the allocation of the intervention sequences.

### Enrollment in hospital

A surveillance system was established in the maternity ward of all five major hospitals in the island. Female hospital supervisors worked in shifts at the maternity ward to cover all deliveries occurring from 7 AM till 8 PM at night. Deliveries occurring after 8 PM were enrolled the next morning. After birth the study team, comprised of hospital staff and study supervisor, screened the newborn for eligibility to participate in the study. If the newborn was found eligible (not very sick, did not need hospitalization and ICU care and without any congenital malformation eliminating the possibility of CHX intervention), the study procedure and purpose was explained to the mother once she was stable. In case the mother was deemed not fit, it was explained to the nearest kin and their consent to participate was sought. If the consent was obtained, the mother-newborn pair was enrolled in the study. The hospital supervisor then opened the envelope for the enrolled pair which contained information about the intervention sequence and the pack. The supervisor took the intervention pack out and handed it over to the hospital staff to apply on the cord of the baby. The hospital supervisor/hospital staff applied CHX on the cord as per the first method mentioned in the sequence and also demonstrated the application to the mother/caretaker and gave the supply for the next 2 days. On discharge, the hospital supervisor completed discharge slip with detailed information from mother about the place where she will be moving after discharge. The case was then handed over to the respective district in charge for follow-up visits in the community.

### Enrollment in community

MCH/TBA informed the central information system (CIS) for any new births occurring in the community. CIS after getting new birth information organized a conference call between the District In charge, Field Supervisor and MCH staff responsible for that area to plan immediate visit to that household. Field supervisor with MCH staff visited the household of the newborn and took consent from mother. Supervisor then opened the envelope containing the intervention sequence and container for that newborn. MCH staff applied the cleansing solution to the tip, base and stump of newborn’s umbilical cord and Supervisor demonstrated the application to the mother/caretaker and gave the supply for the next 2 days. At enrollment, information was collected on SES (socio-economic status) features, pregnancy history, problems during delivery, birth characteristics, and newborn care practices.

### Follow-up visits

Follow-up home visits were conducted by the MCH staff/study supervisor on day 4, 7 and 10. During the follow-up, mother was asked to put a tick on the log sheet on the days she applied the allocated mode of intervention. On the visit day-4 and −7, MCH staff/study supervisor applied CHX using second and third type of container, respectively (as per the sequence allocated). They also demonstrated the application method for cleaning the cord to the mother and left the containers to be used for next 2 days with the mother. On these visits, data on the reported use of solution by the mother was recorded by checking the log sheet and counting the number of used containers. In case the mother had not applied the CHX, the study team member asked the mother of the reason for not applying the intervention and recorded it in the questionnaire.

### Assessment visit

The household was visited on day 10 for final assessment. The mother was asked about her experience of using different delivery modes for cleaning the cord in terms of convenience and preference for the choice of the container i.e. how easy or difficult it was to use them and her preferred container. The staff also recorded number of days mother used the cleaning solution/gel from the log sheet.

#### TIPs for MCH and hospital staff conducting deliveries

TBA, MCH and hospital staff undertaking the deliveries and involved in the TIPs component of the study were also interviewed regarding their experience and feedback on the three different delivery methods used.

### Chlorhexidine preparations

CHX solution was prepared by Galentic Pharma (India) Pvt. Ltd. It contained chlorhexidine gluconate 20 % *w/v* solution BP, polyoxyl 40 hydrogenated castor oil NF (RH 40), carmoisine, purified water BP, and isopropyl alcohol BP. Chlorhexidine gel contained chlorhexidine gluconate, hydroxyl-propyl methyl cellulose, glycerin, methyl-paraben, propyl-paraben and purified water.

### Statistical analysis

Descriptive statistics (frequencies, percentages, means and standard deviation) were calculated, excluding missing data from the analysis. Convenience and preference scores were calculated based on mothers, MCH’s and hospital staff’s feedback. A container was assigned convenience score of ‘2’ if it was selected as most convenient to use, a score of ‘1’ if it was selected as convenient and a score of ‘0’ if it was difficult to use. Preference scores were assigned based on the preference/choice of container. A score of “2” for first preference; “1” for second preference, and the non-preferred container received a score of ‘0’. Data were analyzed using SPSS Statistical Program Version 18.0 (SPSS, Chicago, IL).

## Results

Among the 204 mother-newborn pairs, 27 were lost to follow up primarily due to families moving out of the area, leaving 177 pairs (87 %) who completed the 10 days follow-up period. 17 % of the pregnant women in this study were over 35 years of age and 24 % were illiterate. More than half of the enrolled women were housewives (Table 1).

**Table 1:** Study participant characteristics (*N* = 204)

Characteristics	N (%)
Age of mother	
≤18 years	8 (3.9)
19–35 years	161 (78.9)
>35 years	35 (17.2)
Literacy	
Mother (Illiterate)	49 (24.0)
Father (Illiterate)	37 (18.1)
Occupation	
Mother (Housewife)	105 (51.5)
Father (Fishing/ Farming)	86 (42.2)
Income	
Mother (None)	123 (60.3)
Father (None/ < 50,000 shilling)	72 (35.3)
Parity	
Primiparous	34 (16.7)
2–3	61 (29.9)
4–8	91 (44.6)
>8	18 (8.8)

### TIPs for different modes of CHX delivery

Allocation of the intervention sequence (three application methods; A-100 ml bottle, B-10 ml dropper bottle and C-gel) to one of the six possible delivery sequences i.e. (A B C) or (A C B) or (B A C) or (B C A) or (C A B) or (C B A) was uniformly distributed. TIPs revealed that in 81 % of the cases, first application of CHX occurred within 12 h of birth and in 72 % cases within 8 h, irrespective of the mode.

The compliance was high; 97 % of mothers used all the three modes of intervention. No adverse event due to any mode of intervention was reported during the course of study. Mothers reported little difficulty in using three application methods (100 ml bottle – 83.1 % reported no difficulties, 10 ml dropper bottle – 89.3 %, Gel – 71.8 %). It was observed that an additional effort was required to apply the gel (15.8 %); 100 ml bottle (10.7 %) and 10 ml dropper bottle (5.1 %). Gel preparation took more time to dry (7.3 %) than the other two application methods.

**Fig. 1:**
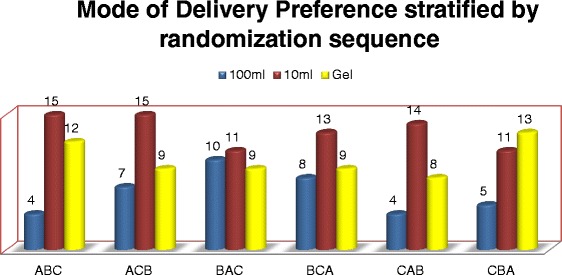
Preference for mode of Chlorhexidine application stratified by randomization sequence. *Values depict numbers

Most of the mothers felt that the 10 ml dropper bottle was most convenient to apply (49.7 %) compared to 100 ml container (19.8 %) or gel tube (32.2 %). From the mothers’ perspective, even though cotton ball made the application easier, single use dropper bottle was more convenient to use than single use gel. Even when convenience scores were calculated, 10 ml single use dropper bottle was found to be more convenient by the mothers/caretakers than 100 ml container or gel tube (Mean convenience score for 10 ml bottle 1.4, in comparison to 0.8 and 0.9 for 100 ml and gel respectively). Mothers/families selected 10 ml dropper bottle (44.6 %) as their most preferred choice over the 100 ml bottle (20.9 %) or gel tube (33.9 %) for cleansing the umbilical cord of the newborn. When the different application methods were compared, the preference score was highest for 10 ml single use dropper bottle (Mean preference score 1.4 as compared to 0.8 and 0.9 for 100 ml and gel respectively- Table 2). Mothers preferring 10 ml single use bottle or gel tube also seem to be well aware of good newborn care practices (Table 3). Based on the preference score, TBAs, medical staff and CHWs preferred 10 ml dropper bottle (43.3 %) over 100 ml bottle (12.9 %) and gel (38.8 %). Delivery sequence did not change the preference for mode of delivery (Fig. 1).

**Table 2:** Preference and convenience scores

Preference Scores	100 ml	10 ml	Gel
Mothers			
Most preferred (Score 2)	37 (20.9)	79 (44.6)	60 (33.9)
Less preferred (Score 1)	60 (33.9)	69 (39.0)	48 (27.1)
Not preferred (Score 0)	80 (45.2)	29 (16.4)	60 (39.0)
Mean scores for preference ± (SD)	0.8 (0.7)	1.4 (0.7)	0.9 (0.8)
MCH Workers			
Most preferred (Score 2)	12 (17.9)	26 (38.8)	29 (43.3)
Less preferred (Score 1)	11 (16.4)	30 (44.8)	26 (38.8)
Not preferred (Score 0)	44 (65.7)	11 (16.4)	12 (17.9)
Mean scores for preference ± (SD)	0.5 (0.8)	1.2 (0.7)	1.2 (0.8)
Convenience Scores			
Mothers			
Most preferred (Score 2)	35 (19.8)	88 (49.7)	57 (32.2)
Less preferred (Score 1)	77 (43.5)	66 (37.3)	53 (29.9)
Not preferred (Score 0)	65 (36.7)	23 (13.0)	67 (37.9)
Mean scores for convenience ± (SD)	0.8 (0.7)	1.4 (0.7)	0.9 (0.8)
MCH Workers			
Most preferred (Score 2)	10 (14.9)	31 (46.3)	32 (47..8)
Less preferred (Score 1)	40 (59.7)	35 (55.2)	25 (37.3)
Not preferred (Score 0)	17 (25.4)	1 (1.5)	10 (14.9)
Mean scores for convenience ± (SD)	0.9 (0.6)	1.4 (0.5)	1.3 (0.7)

All figures shown are N(proportion) unless otherwise indicated

**Table 3:** Newborn care practices and mode of Chlorhexidine application preference

Practices	100 ml	10 ml	Gel
Thermal care provided (*N* = 108)	20 (18.6)	44 (40.7)	44 (40.7)
Skin to skin contact (*N* = 158)	32 (20.3)	73 (46.2)	53 (33.5)
Child wrapped in clean cloth (*N* = 174)	38 (21.8)	76 (43.7)	60 (34.5)
Did not bath baby immediately after birth (*N* = 152)	31 (20.4)	73 (48.0)	48 (31.6)
Fed on colostrum (*N* = 153)	36 (23.5)	67 (43.8)	50 (32.7)

Figures shown are N(%)

N indicates the number of mothers/caregivers providing newborn care practice

## Discussion

Chlorhexidine is an inexpensive, safe and effective cord care intervention for reducing neonatal morbidity and mortality in low-resource settings [13–15]. The present study evaluated the acceptance and impediments to using chlorhexidine comparing three different modes (3 different packaging: 100 ml bottle with cotton swab, 10 ml single use dropper bottle and 3 gm single application gel tube) of delivery for cord cleaning in terms of acceptance, ease of use and effectiveness in covering the target area using TIPs (Trials of Improved Practices) methodology. The overall acceptability in terms of convenience and preference was high for 10 ml single use dropper bottle; which was liked by most of the mothers, TBA/MCH and hospital staff over 100 ml bottle and gel tube. Despite chlorhexidine (in liquid form) being spread over the abdomen through its use, 10 ml single use dropper bottle was the preferred choice. Mothers did not find much difficulty in applying the solution. The advantage of using a crossover design was that every mother had an experience of testing all the three modes of delivery of chlorhexidine to apply on the umbilical cord and could therefore perceive the risks/benefits associated with each.

There were no apparent side-effects and no serious adverse events related to any of the interventions used during the course of study. The concentration of chlorhexidine used was equivalent to that used in prior trials [13–15, 17, 26, 27]. All the three modes had the same concentration of chlorhexidine (4 %) and the gel formulation was thickened using hydroxy propyl methyl cellulose. Both the gel and liquid formulations were produced by Galentic Pharma (India) Pvt. Ltd. and made available at a low cost of ~ USD 0.02 per application.

Two studies conducted in Nepal which evaluated the acceptability and ease of use of gel and liquid chlorhexidine indicated that gel formulation was more acceptable and a preferred approach by families over liquid formulation [16, 17]. However, no information on the choice of delivery container (100 ml bottle with cotton swab or 10 ml dropper bottle) was provided for chlorhexidine liquid solution application. There can also be a possibility in those studies that the participants failed to express negative concerns about the intervention, anticipating better care. In previously conducted trials in Nepal, Bangladesh and Pakistan, chlorhexidine was applied using wipes, cotton balls or syringes [15, 17, 28, 29].

Information collected through TIPs helped in selection and implementation of a culturally acceptable intervention for the main trial ‘evaluating the efficacy of use of chlorhexidine to clean umbilical cord of neonates in first 10 days for reduction in neonatal mortality and omphalitis (Clinical Trial Number: ClinicalTrials.gov NCT01528852)’. Based on the choice of mothers, hospital staff and TBA/MCH, the 10 ml single use dropper bottle was selected for the RCT.

## Conclusion

It is the first trial of its kind reporting mothers/caretakers and health professionals’ acceptability and preference for various possible modes of chlorhexidine delivery for cord care. 10 ml single use dropper bottle was given highest preference for delivery of intervention. In wake of current effort to scale up chlorhexidine cord care interventions in various countries, with appropriate changes in WHO recommendation for cord care, our pilot study has lot of relevance for the programs intending to implement chlorhexidine interventions for reduction in omphalitis and neonatal mortality. Selection of the most acceptable method of intervention delivery is essential to the design and implementation of the intervention efficacy trials as well as successful implementation of programs.

## Abbreviations

CHX: Chlorhexidine
TIPs: Trials of Improved Practices
TBA: Trained birth attendants
MCH: Maternal and child health
SES: Socio-economic status
SPSS: Statistical Package for the Social Sciences
